# Electrical Treatment of Nævi

**Published:** 1910-06-04

**Authors:** 


### Dermatology.

### ELECTRICAL TREATMENT OF NÆVI.

An important paper by Dr. H. L. Jones on the Treatment of Nævus by Electrical Methods, is published in the *Archives of the Roentgen Ray*. The paper was read before the Electro-Therapeutic Section of the Royal Society of Medicine, and is based upon an analysis of 1,600 cases, more than two-thirds of which were in the female sex. Whether this disparity is due to the greater interest of parents in the cosmetic appearance of girl babies, or to a real disparity of incidence, the author does not discuss. About half of these nævi occurred upon the face or head, a quarter on the trunk, and an eighth on the limbs. It is noted that the midline is a frequent site, especially on the scalp, face, and perineum; such median nævi often derive blood from the arteries of both sides. For solid nævi when not on the face, and button-shaped nævi with raised edges, the author favours excision; but for all others he prefers electrical methods. No age is too tender for such treatment; and since nævi, almost without exception, increase in size, the eradication of all nævi should be undertaken as soon as possible after their discovery. The only exception admitted to this rule is the superficial nævoid condition sometimes seen on the forehead or nape of the neck at birth, consisting in a thin uniform redness of considerable extent: this pseudo-nævoid condition calls for no treatment, as it generally disappears spontaneously in a few months.

### Port-wine Stains.

Port-wine stains Dr. Jones finds especially amenable to treatment, and much less liable to recurrence than true nævi. The bright-red florid nævus, so common in babies a few weeks old, on the other hand, is frequently refractory, often recurs, and requires to be very carefully and thoroughly dealt with. Nævi on the fingers, *per contra*, must not be treated too vigorously; for the blood supply to the digits is feeble in babies, and too energetic an application may result in sores which take a very long time to heal up.

The electrical methods of treatment useful in nævus cases are five in all, including electrolysis, the galvano-cautery, *x*-rays, radium, and high-frequency applications. Of these, the galvano-cautery is recommended as that best suited as a routine method, though each of the others is of use in special cases. The galvano-cautery point should be as fine as possible, and should be used to produce vertical punctures all over the nævus, about $\frac{1}{6}$ in. apart in the centre and rather more closely towards the periphery. The thickness of the growth must be estimated, and the treatment—whether electrolysis or cautery is being used—should reach through the whole depth of it. On the scalp the nævoid condition not rarely extends right down to the pericranium, and it is a good plan to puncture until the point actually impinges on the skull. The art of treating nævi so as to leave the minimum of scarring lies in knowing how much can be attempted at a time: to do too much may obliterate the nævus more rapidly, but leave a most objectionable scar.

### Electrolysis.

In performing electrolysis there are two permissible methods. In one a number of needles alternately of opposite polarity are fixed into one handle, which keeps them parallel and equalises the current along their whole extent: platinum needles alone can be used in this way. The other plan is to plunge one needle only into the growth, completing the circuit through the patient's body by a moist pad. In this way a zinc needle can be used and connected with the positive pole of a battery, and metallic ions can thus be deposited in the substance of the nævus. A smaller current is needed than when using a number of platinum needles; and if the needle used be copper, great care is needed to avoid setting up a condition of sloughing.

For port-wine stain *x*-rays and radium have been found valuable: but the treatment by mild fulguration with a high-frequency current after the manner of Dr. Bergonié is highly recommended. The applications need to be renewed, as a rule, at intervals of about three weeks, and are said to be very successful in the cure of this particular nævoid disfigurement. The method has the advantage over *x*-rays and over radium of not being liable to produce telangiectases.

